# Very Preterm Infants Failing CPAP Show Signs of Fatigue Immediately after Birth

**DOI:** 10.1371/journal.pone.0129592

**Published:** 2015-06-08

**Authors:** Melissa L. Siew, Jeroen J. van Vonderen, Stuart B. Hooper, Arjan B. te Pas

**Affiliations:** 1 The Ritchie Centre, MIMR-PHI, Monash University, Clayton, Australia; 2 Division of Neonatology, Department of Pediatrics, Leiden University Medical Center, Leiden, the Netherlands; Hôpital Robert Debré, FRANCE

## Abstract

**Objective:**

To investigate the differences in breathing pattern and effort in infants at birth who failed or succeeded on continuous positive airway pressure (CPAP) during the first 48 hours after birth.

**Methods:**

Respiratory function recordings of 32 preterm infants were reviewed of which 15 infants with a gestational age of 28.6 (0.7) weeks failed CPAP and 17 infants with a GA of 30.1 (0.4) weeks did not fail CPAP. Frequency, duration and tidal volumes (VT) of expiratory holds (EHs), peak inspiratory flows, CPAP-level and FiO_2_-levels were analysed.

**Results:**

EH incidence increased <6 minutes after birth and remained stable thereafter. EH peak inspiratory flows and VT were similar between CPAP-fail and CPAP-success infants. At 9-12 minutes, CPAP-fail infants more frequently used smaller VTs, 0-9 ml/kg and required higher peak inspiratory flows. However, CPAP-success infants often used large VTs (>9 ml/kg) with higher peak inspiratory flows than CPAP-fail infants (71.8 ± 15.8 vs. 15.5 ± 5.2 ml/kg.s, p <0.05). CPAP-fail infants required higher FiO_2_ (0.31 ± 0.03 vs. 0.21 ± 0.01), higher CPAP pressures (6.62 ± 0.3 vs. 5.67 ± 0.26 cmH_2_O) and more positive pressure-delivered breaths (45 ± 12 vs. 19 ± 9%) (p <0.05)

**Conclusion:**

At 9-12 minutes after birth, CPAP-fail infants more commonly used lower VTs and required higher peak inspiratory flow rates while receiving greater respiratory support. VT was less variable and larger VT was infrequently used reflecting early signs of fatigue.

## Introduction

Continuous positive airway pressure (CPAP) is progressively replacing intubation and mechanical ventilation as the first choice of respiratory support for premature infants at birth. However a considerable number of infants, who are initially stabilised with CPAP, will develop worsening respiratory failure and eventually require intubation for mechanical ventilation and the administration of surfactant.[1–6] If early identification of preterm infants who fail CPAP is possible this could be used to optimise the timing of surfactant treatment to early administration (within 2 hours) [7], decrease pulmonary damage and improve outcomes.

Many measurements have been investigated for their ability to predict CPAP failure in infants such as FiO_2_, PaO_2_, A-aDO_2_, a/A ratio, PaO_2_/FiO_2_ and the stable micro bubble test as soon as possible after birth.[8–13] To date, the respiratory pattern and effort at birth have not been investigated although they are a major determinant of lung gas volumes [14] and may correlate with respiratory distress severity and predict CPAP failure.[15] As the preterm infant’s chest wall is highly compliant and transiently expands immediately after birth [16], it has limited capacity to oppose lung recoil.[17] As such, these infants must utilise their breathing pattern to develop and maintain functional residual capacity (FRC) immediately after birth.

Expiratory holds (EHs), which are breaths characterised by a period of zero flow during expiration and prolonged duration of expiratory [18–20], help to maintain FRC.[14,18,21] Studies in newborn rabbits, lambs and infants suggest that the use of EHs is influenced by changes in lung gas volumes and airway pressure.[14,19,22,23] Although it is uncertain whether EHs can indicate absolute lung gas volumes, we have previously shown a strong relationship between the incidence of EHs and FRC in newborn rabbits.[14]

Large tidal volumes (VTs) at birth promote lung liquid clearance by generating transpulmonary pressures that move liquid from the airspace into the peri-alveolar interstitial tissue.[14,16] There is a positive relationship between VT and FRC with large VTs resulting in larger immediate FRC accumulation in the first breaths after birth.[24] A similar relationship is observed regarding inspiratory effort and FRC development.[24] Clearly, VT and inspiratory flow can influence end-expiratory lung gas volumes.

As preterm infants are commonly surfactant deficient and have a highly compliant chest, they commonly have lower than normal resting lung gas volumes.[25,26] It is possible that infants with the lowest lung gas volumes represent those most likely to develop severe respiratory distress syndrome (RDS) and thus require more respiratory support than CPAP initiated at birth. Indeed, up to 80% of infants who fail CPAP demonstrate moderate-severe RDS.[1,27] As such, preterm infants who fail to establish a good breathing pattern and effort immediately after birth are more likely to be unable to maintain their FRC and eventually require increased respiratory support. We hypothesized that the breathing pattern and effort at birth is different in infants who failed CPAP within 48 hours after birth than in infants where CPAP was successful.

## Methods

The local institutional review boards (IRBs) of the Leiden University Medical Center (Commissie Medische Ethiek, Leids Universitair Medisch Centrum) and Royal Women’s Hospital (the Human Research Ethics Committee, Royal Woman’s Hospital) approved physiological- and video recordings at birth in the delivery room when respiratory support was necessary for research purposes. Written parental consent to use the recordings for research was obtained after birth. For this retrospective observational study respiratory function recordings made between years 2009 and 2011 of infants born <32 weeks of gestation who were supported with CPAP at birth were included. The aim of this study was to determine if the breathing pattern and effort at birth could predict which infants could be stabilised with CPAP in the delivery room but later require intubation. Therefore, infants that were intubated in the delivery room were excluded from the analysis.

Resuscitation was performed by neonatologists, neonatal fellows or pediatric registrars who used a T-piece infant resuscitator (Neopuff; Fisher & Paykel Healthcare, Auckland, New Zealand) in combination with a Laerdal silicone round mask of an appropriate size (Laerdal, Stavanger, Norway). In all infants included in this study positive pressure ventilation was performed according to Dutch guidelines starting with 5 initial sustained inflations, each lasting 2–3 seconds, a peak inspiratory pressure (PIP) of 20 cmH_2_O, a positive end expiratory pressure (PEEP) of 5 cmH_2_O and a gas flow rate of 8 L/min using air.[28] Positive pressure ventilation was continued if spontaneous breathing was absent or if the infant’s heart rate and oxygen saturation were below the levels described in the Dutch guidelines.[29] Otherwise, infants were supported with 5–6 cmH_2_O of CPAP.

Respiratory interventions were recorded starting from birth, e.g. when the head is delivered, using a webcam and a Florian respiratory function monitor (Acutronic Medical Systems AG, Hirzl, Switzerland), with a hot wire anemometer as a flow sensor between the T-piece and facemask (dead space <1 ml) to detect gas flow in and out of the mask. The flow signal was integrated to measure inspired and expired tidal volumes (V_Ti_ and V_Te_) and the difference equals mask leak ((V_Ti_—V_Te_/ V_Ti_)*100).[30] The flow sensor was calibrated before each recording. Data recording was started at the exact time of birth (when the shoulders are delivered). Pressure was measured from the distal section of the T-piece tubing. Oxygen saturation and heart rate were measured with a Masimo SET pulse oximeter (Masimo Radical, Masimo Corporation, Irvine, California). FiO_2_ was measured using an Oxylog (Teledyne technologies, thousand oaks, California). Signals of gas flow, VT, ventilatory pressure, FiO_2_, oxygen saturation, heart rate and breathing were digitised and recorded at 200 Hz using Spectra software (Grove Medical, Hampton, UK).

The resuscitators were not blinded to the respiratory monitor, but we recently reported that they rarely used the monitor for feedback.[28] The researcher performing the recording was not part of the resuscitation team and did not inform the resuscitators of the saturation, heart rate and respiratory function of the infant displayed by the monitor.

Respiratory function recordings were analysed in 3-minute periods to determine the frequency of EHs, the volume-time integral of EHs (i.e. area under the volume recording of each EH), EH hold duration and EH hold volume. All spontaneous breaths, EHs and other breaths, were analysed for tidal volume (VT) and peak inspiratory flow. The volume-time integral of each EH takes into account the remaining lung gas volume and the size and duration of the EH, thus providing an overall measurement of gas exchange potential. In order to take a spontaneous breath, the inspiratory muscles must generate sufficient force to overcome the elasticity of the lungs and chest wall therefore we used peak inspiratory flow rate as a surrogate to determine of respiratory strength. CPAP pressure and FiO_2_ before the infant was transferred to the neonatal intensive care unit (NICU) was also noted. To blind investigators, if the infant was intubated, the eventual time of intubation was added to the database after the analysis was finished. The threshold for CPAP failure was intubation within 48 hours of age because intubation within this time was most likely due to respiratory distress rather than apnoea’s of prematurity or the presence of infection.

Indications for endotracheal intubation in the delivery room were (1) inability to maintain SpO_2_ in the target range (85–95%) with a maximum CPAP pressure of 8 cmH_2_O and (2) FiO_2_ >0.4. Surfactant was only administered after the infants was intubated. Our local guidelines did not specify the time point at which tracheal intubation had to be performed. Indications for endotracheal intubation the first 48 hours after birth included at least one of the following; (1) inability to maintain SpO_2_ in the target range (85–95%) with a maximum CPAP pressure of 8 cmH_2_O and FiO_2_ >0.4, (2) more than one apnea per hour for 6 hours despite caffeine treatment or any apnea requiring PPV or (3) a respiratory acidosis (pH <7.25 and pCO_2_ >60 mmHg and rising) on two separate blood gases. Caffeine was provided to all infants shortly after admittance to the NICU. CPAP was provided using binasal prongs and nasal masks which are switched every 12 hours to prevent pressure sores. Corticosteroids were given before birth in two gifts with 48 hours in between.

Results are presented as mean ± standard error of the mean or otherwise stated. Data were tested for normality and equal variance and data transformations were performed if necessary using SigmaPlot (SigmaPlot 12, Systat Software Inc. Chicago, Illinois). A repeated measures ANOVA and a Student-Newman-Keuls post hoc test was performed to identify statistical differences. A p-value of <0.05 was used for statistical significance. When Student’s t-tests were used to compare groups, data that were not normally distributed were analysed using a Mann-Whitney Rank Sum test.

## Results

72 respiratory recordings were eligible for analysis. In total 40/72 recordings needed to be excluded for the following reasons: 14 respiratory recordings were of poor quality (excessive movement artefact, significant leak from the facemask or problems with the digitisation of data), 9 infants lacked enough spontaneous breaths to be analysed (<10 spontaneous breaths in the entire recording), for 7 infants the corresponding patient data could not be located, 7 infants were intubated in the delivery room and 3 infants died during the initial resuscitation period. Therefore, a total of 32 respiratory recordings were analysed; 15 recordings of CPAP-failed infants and 17 recordings of CPAP-success infants. The patient characteristics of these groups are presented in Table 1. Infants that did not receive a full course of steroids received all received one gift. It was not expected that the same number of recordings were analysed at each time point in the analysis because periods of IPPV were excluded and the duration of assisted ventilation in the delivery room differed between patients. Table 2 presents the number of patients included in each time point.

**Table 1:** Patient details.

		CPAP fail	CPAP success
Total patients		15	17
Gestational age (weeks)		28.6 ± 0.7	30.1 ± 0.4*
Weight (g)		1090 ± 140	1270 ± 80*
Gender	Male	6	12
	Female	9	5
Mode of delivery	C-section	9	8
	Vaginal	6	9
Received antenatal steroids (full course)		9	6
Time of intubation (hours)		10.62 ± 2.38	0

**Table 2:** The number of patient recordings analysed in each time period.

Time period (min)	0–3	3–6	6–9	9–12
CPAP fail (n = 15)	4	12	13	10
CPAP success (n = 17)	8	16	17	7

Infants who failed CPAP required more inflations at birth (45 ± 12% of all flow waves) compared to infants who were successfully supported by CPAP (19 ± 9% of all flow waves; p<0.05). Infants who failed CPAP within 48 hours were intubated, on average, at 6.62 ± 0.31 hours after birth. In the CPAP-fail group, 1999 spontaneous breaths were analysed. In the CPAP-success group, 2004 spontaneous breaths were analysed.

**Fig 1:**
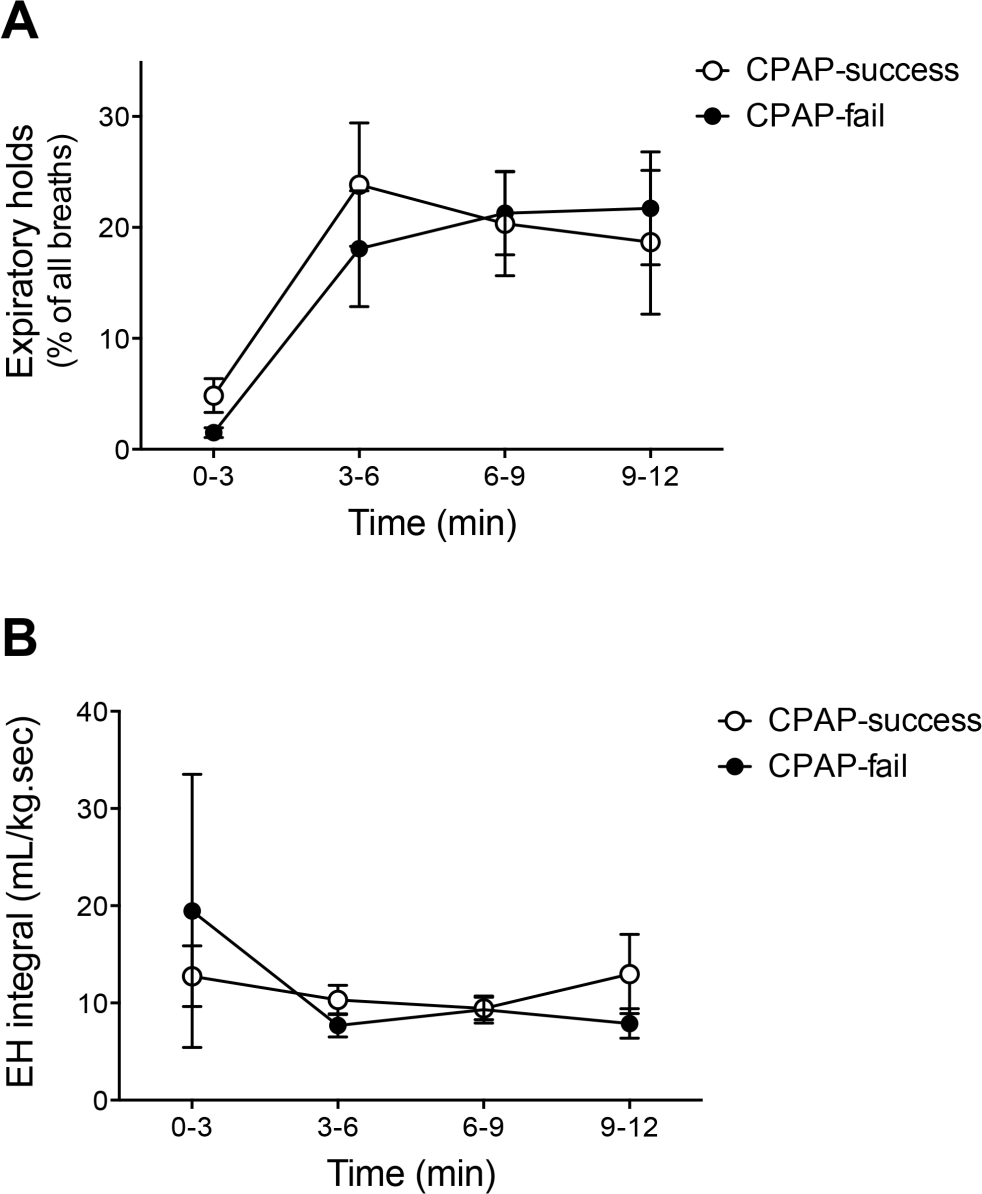
The frequency of EHs (A) and EH integral (B) in preterm newborns in the first 12 minutes after birth. Open circles are CPAP success newborns. Closed circles are CPAP fail newborns.

In all infants, the incidence of EHs increased from <5% at 0–3 minutes to ~20% at 3–6 minutes after birth (p<0.05) and remained stable at 6–9 minutes and at 9–12 minutes (Fig 1A). There were no significant differences between CPAP-fail and CPAP-success groups at any time point (p>0.05). The volume-time integral of breaths exhibiting an EH was similar between CPAP-fail and CPAP-success groups at each of the selected time points after birth (p>0.05, Fig 1B).

**Fig 2:**
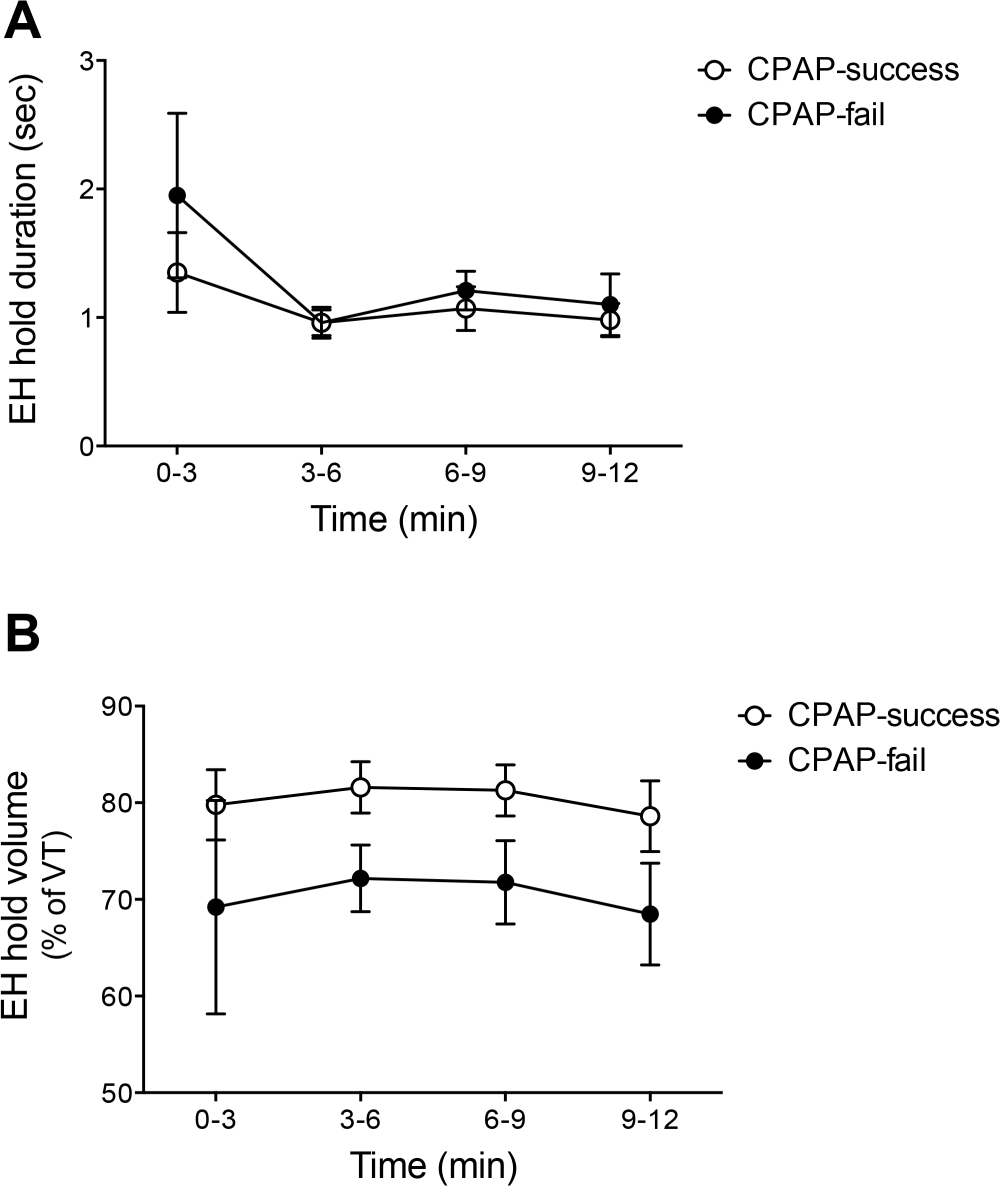
The duration (A) and percentage of gas volume in the lungs (B) during the period of zero flow of EHs in preterm newborn in the first 12 minutes after births. Open circles are CPAP success newborns. Closed circles are CPAP fail newborns.

The duration of the period of zero flow occurring during the EH was not different between CPAP-fail and CPAP-success groups (p>0.05; Fig 2A). The average volume of gas in the lungs during the hold was also not statistically different (p>0.05), although the CPAP success group tended to maintain a greater volume of gas in the lungs than the CPAP fail group (Fig 2B).

**Fig 3:**
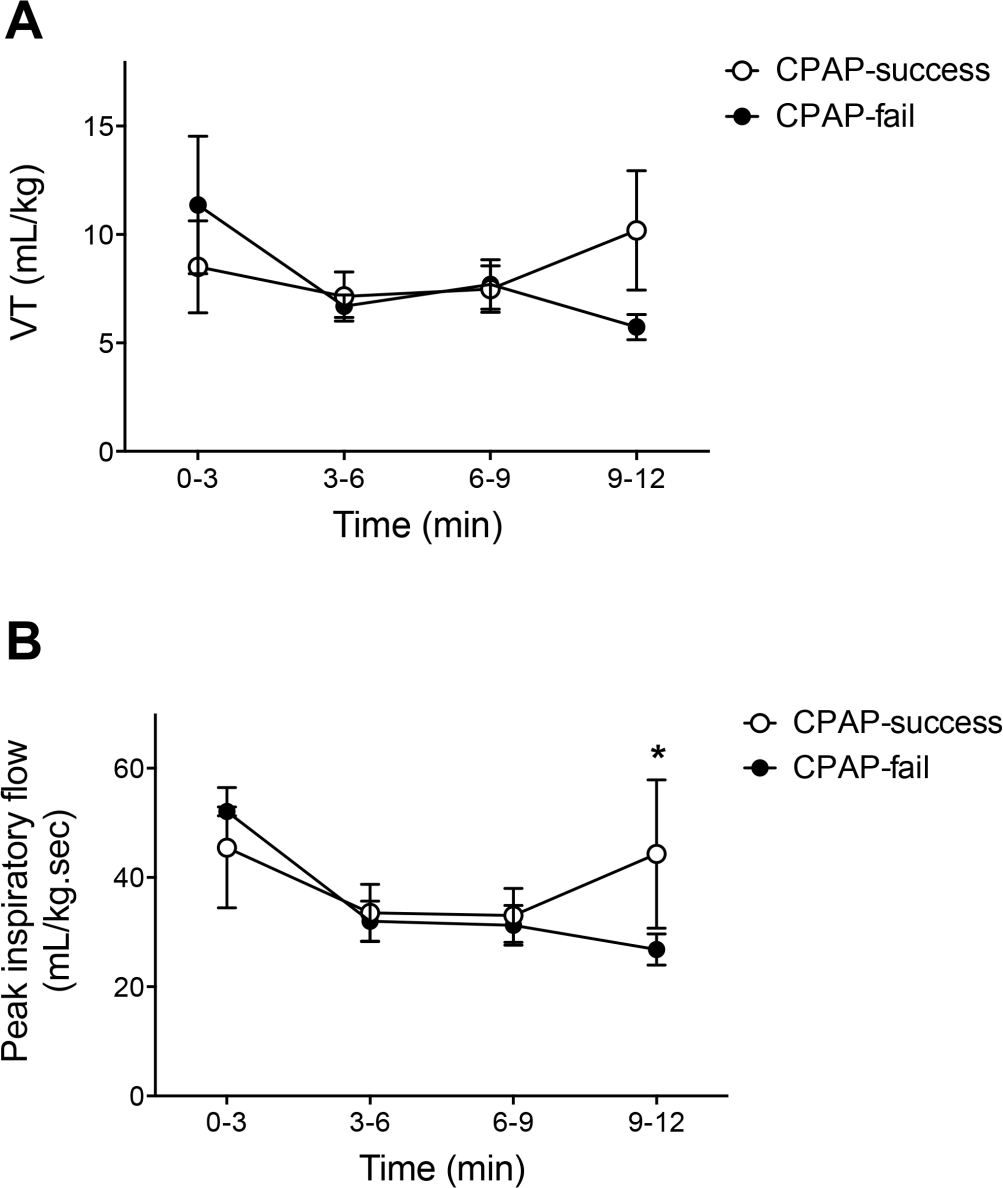
The VTs (A) and peak inspiratory flow (B) of all breaths generated by preterm newborns in the first 12 minutes after birth. Open circles are CPAP success newborns. Closed circles are CPAP fail newborns.

The VT of all spontaneous breaths was not different between CPAP-fail and CPAP-success groups at any time point (p>0.05, Fig 3A). However, at 9–12 minutes after birth, the CPAP-fail infants had relatively consistent VTs (median [IQR]) (6.3 [4.4–6.9]) whereas VTs within the CPAP-success group demonstrated greater variability (7.5 [5.3–16.4]). The coefficient of variation of VTs at 9–12 minutes in the CPAP-fail group was 0.32, whereas it was 0.66 in the CPAP-success group. Similar to VT, peak inspiratory flow averaged across all spontaneous breaths was not different between CPAP-fail and CPAP-success groups at most time points (p>0.05). However, at 9–12 minutes CPAP-fail infants demonstrated significantly lower peak inspiratory gas flows than CPAP-success infants (p<0.05; Fig 3B). The peak inspiratory flows at 9–12 minutes were less variable in the CPAP-fail group (29.5 [21.0–35.4] mL/kg/sec) than in the CPAP-success group (33.0 [16.5–54.9] mL/kg/sec). The coefficient of variation was 0.35 in the CPAP-fail group and 0.82 in the CPAP-success group.

**Fig 4:**
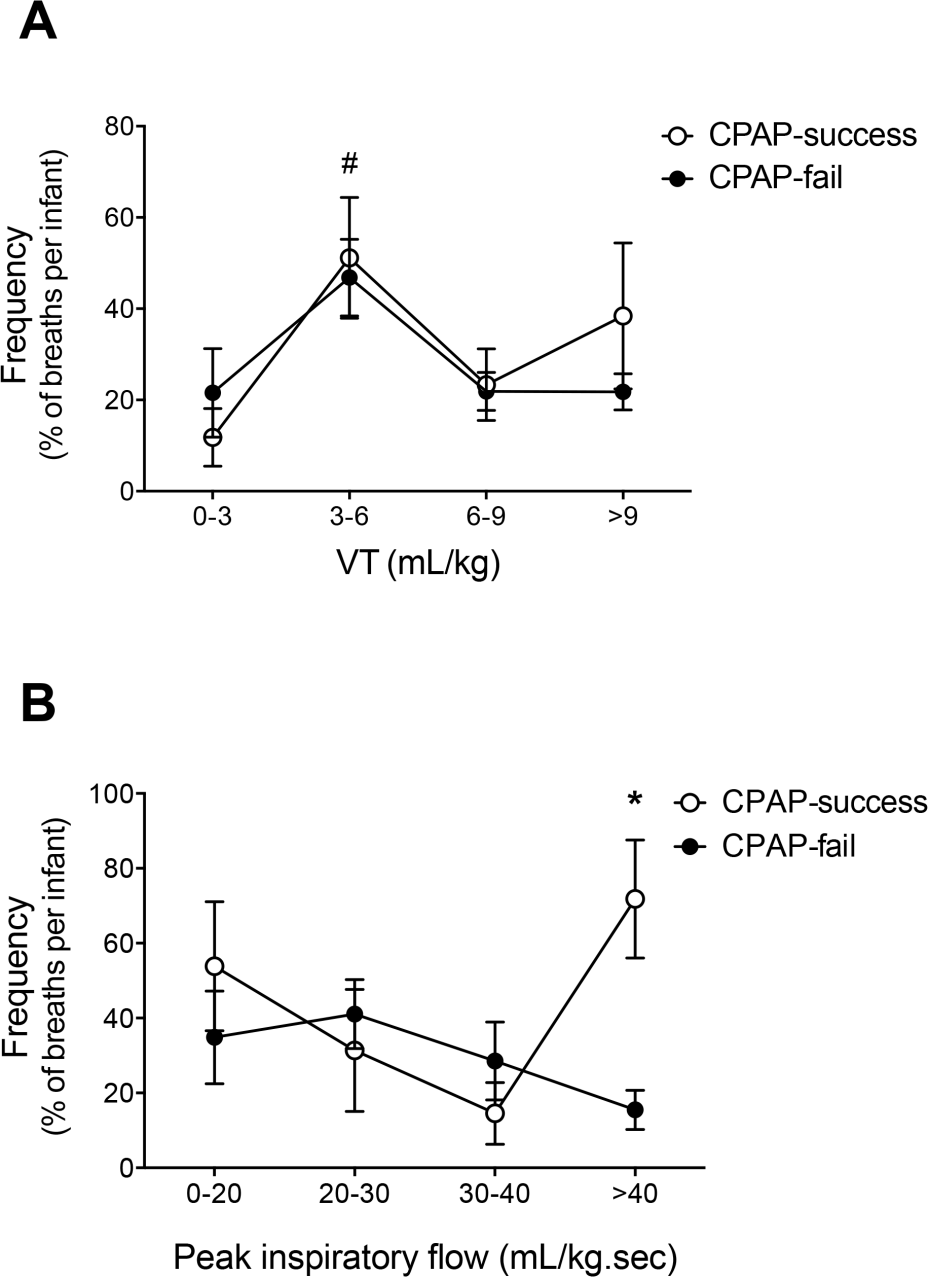
The frequency of different VTs (A) and peak inspiratory flows (B) of all breaths generated by preterm newborns in the first 12 minutes after birth. Open circles are CPAP success newborns. Closed circles are CPAP fail newborns. # indicates that the VT range is significantly greater than any other VT range in both CPAP-success and CPAP-fail infants. * indicates that the frequency of the CPAP-success group is significantly different to the CPAP-fail group at the corresponding peak inspiratory flow.

Breaths occurring between 9–12 minutes were analysed in individual infants to determine the usage of different sized VTs and peak inspiratory flows in CPAP-fail and CPAP-success infants at this time. CPAP-fail and CPAP-success infants most commonly utilised VTs between 3–6 mL/kg at 9–12 minutes after birth (Fig 4A; p<0.05). Although not significant, CPAP-success infants appeared to utilise almost 2 times as many VTs of >9 mL/kg than CPAP-fail infants. However, CPAP-success infants achieved large inspiratory flows >40 ml/kg.s ~4 times more frequently than CPAP-fail infants (15.5 ± 5.2% vs. 71.8 ± 15.8%, p<0.05) (Fig 4B). In CPAP-success infants 70% of the very large VTs (>9 mL/kg) were generated by using large inspiratory flows >50 mL/kg.s. In contrast, CPAP-fail infants less commonly used VTs >9 mL/kg and utilised a wide range of inspiratory flows to achieve these volumes.

**Fig 5:**
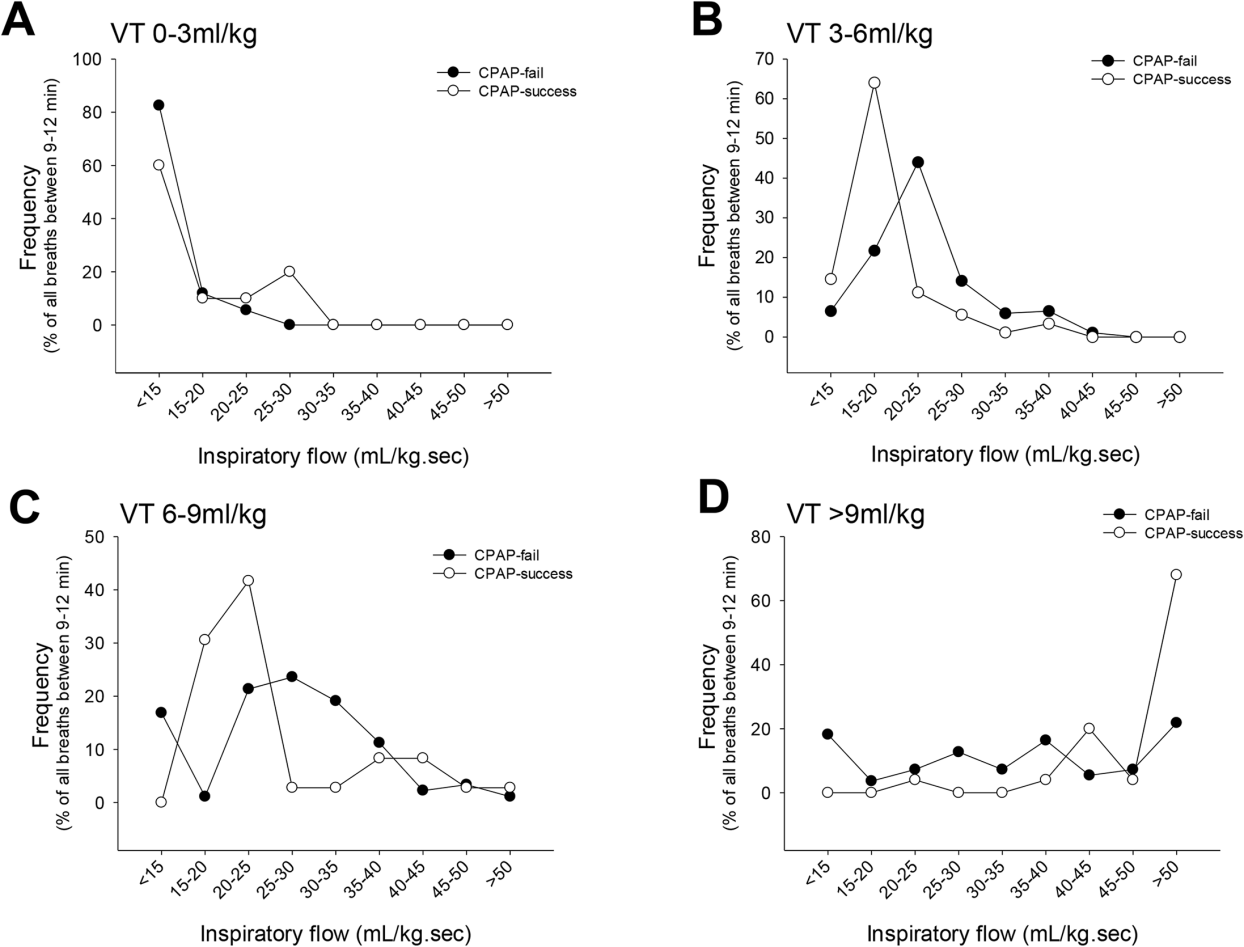
An analysis of breaths generated between 9–12 minutes after birth separated into different VT ranges; VT 0–3 mL/kg (A), 3–6 mL/kg (B), 6–9 mL/kg (C) and >9 mL/kg (D). Each graph shows the frequency of different peak inspiratory flows of all breaths within the specific VT range. Open circles are CPAP success newborns. Closed circles are CPAP fail newborns.

At 9–12 minutes after birth breaths were separated into VT ranges and the distribution of peak inspiratory flows determined (Fig 5). Low VTs (0–3 mL/kg) were mostly achieved with slow peak inspiratory flows <15 mL/kg.s in both CPAP-success and CPAP-fail infants. However, VTs of between 3–6 ml/kg, which was the most commonly utilised VT at 9–12 minutes, were achieved with lower flow rates in CPAP-success infants than CPAP-fail infants (Fig 5). Larger VTs (6–9 mL/kg) were commonly achieved with inspiratory flows of 15–25 mL/kg.s in the CPAP-success group whereas the same VTs required much higher inspiratory flows of 20–35 mL/kg.s in the CPAP-fail group.

**Fig 6:**
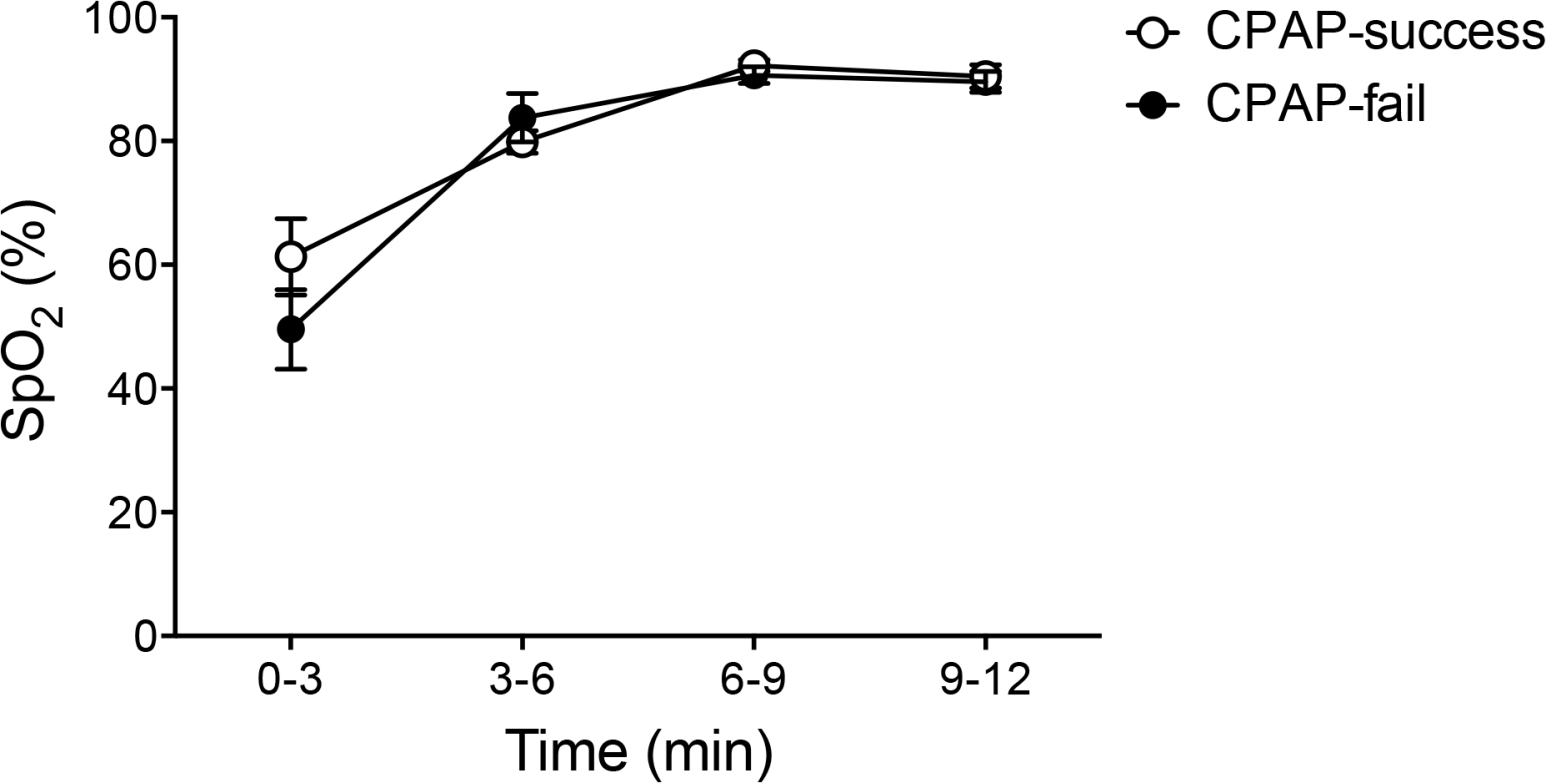
Oxygen saturation changes in the first 12 minutes after birth in preterm newborns. Open circles are CPAP success newborns. Closed circles are CPAP fail newborns.

SpO_2_ increased similarly in CPAP-fail and CPAP-success infants (Fig 6). To achieve these saturations, CPAP-fail infants required greater FiO_2_ (0.31 ± 0.03 vs. 0.21 ± 0.01; p<0.05) and CPAP levels (6.62 ± 0.3 cmH_2_O vs. 5.67 ± 0.26 cmH_2_O, p<0.05) than CPAP-success infants.

## Discussion

Several randomised controlled trials have demonstrated that preterm infants can be stabilised with CPAP at birth.[2–6] However, a significant proportion of these infants, as many as 50% [2,6], developed respiratory failure and failed CPAP. Our study investigated if the breathing pattern at birth could identify infants that fail CPAP within 48 hours after birth. EH frequency, size and duration over time and average VT and average peak inspiratory flow of all breaths did not predict CPAP failure. However, a sub-analysis of breaths occurring between 9–12 minutes demonstrated that the CPAP-fail infants needed higher inspiratory flows to reach the volumes but used very few breaths of large peak inspiratory flows when compared to CPAP-success infants. This reflects more difficulty in breathing and early signs of fatigue in the CPAP-fail infants. Indeed, infants who failed CPAP required greater respiratory support, such as higher FiO_2_ levels, higher CPAP pressures and more positive pressure-delivered breaths, before transport to the NICU.

Low VTs and inspiratory flows have been associated with a greater risk of extubation failure in mechanically ventilated children 3–5 years old.[31] Although our results demonstrated a tendency for CPAP-fail infants to have lower VT at 9–12 minutes, this was not statistically different because of the large variation in VTs of the CPAP-success group. However, peak inspiratory flows were significantly lower in CPAP-fail versus CPAP-success infants and both the VTs and peak inspiratory flows were less variable. Mechanically ventilated preterm infants, studied at ~5 days postnatal age, were most likely to fail extubation if they demonstrated less variation in their mean inspiratory flow rate during a 3 minute spontaneous breathing trial before extubation.[32] This is similarly observed in adults.[33] The coefficient of variation of the VT and peak inspiratory flow at 9–12 minutes suggests that infants who failed CPAP utilised a narrower range of VT and flows than CPAP-success infants.

Large variability reflects the infant’s ability to adapt to a changing lung gas volume to maintain adequate lung aeration. A limited range of breaths (VTs and inspiratory flows) may indicate increased difficulty in maintaining lung aeration. These infants are likely at greater risk of later respiratory failure. Indeed, CPAP-fail infants required greater respiratory support such as increased FiO_2_, greater CPAP pressures and more manually delivered breaths, to obtain similar oxygen saturations to CPAP-success infants. A need for greater respiratory support by infants that fail CPAP has been previously reported.[8,10,11] As described earlier in the NICU we use both binasal prongs and bubble CPAP devices which were shown to be equally effective in achieving desired bubble CPAP pressures.[34]

Maintaining lung aeration is particularly difficult for newborn infants because insufficient mineralisation of the ribs leads to a highly compliant chest wall [35] that tends to collapse inwards under the lungs natural recoil and particularly during inspiration. This effect is more pronounced in preterm infants [36] who have higher chest wall compliances than term infants [17]. Employing faster inspiratory flows to generate larger potential VTs in anticipation that a proportion of the VT will not be achieved could compensate for the loss of potential VT, resulting from chest wall distortion. By increasing their efforts for each breath, CPAP-fail infants can achieve adequate VTs and delay the onset of respiratory distress until hours after birth. Our study demonstrated that CPAP-fail infants required larger inspiratory flow rates to achieve VTs of 3–9 mL/kg than CPAP-success infants. These findings suggest that infants most likely to fail CPAP may be those with the most compliant chest walls, high lung recoil and low muscular strength. In fact, high Silverman scores, which indicate these properties, have been associated with CPAP failure.[11]

In contrast to the observations made at VTs between 0–9 ml/kg, larger VTs >9 ml/kg were more often achieved by CPAP-success infants using higher peak inspiratory flows than CPAP-fail infants (Fig 5D). This observation may identify the strongest infants who are capable to generate the highest inspiratory flows to achieve these larger volumes and therefore are less probable to develop RDS. In addition, CPAP-success infants may have stiffer chest walls, which may help them generate rapid peak inspiratory flows and more easily achieve large recruitment breaths. Although not statistically significant, larger VTs >9 ml/kg, on average, appeared to make up a larger percent of the breathing pattern in CPAP-success infants than fail infants (Fig 5). CPAP-fail infants, possibly with more compliant chest walls, utilised a wider range of inspiratory flow rates to achieve the large VTs of >9 mL/kg (Fig 5). The use of slower rates of inflation to achieve large VTs likely reflects a mechanical problem and a greater difficulty in simply moving air into the lungs. This suggestion is supported by the finding that these infants required higher flow rates to achieve moderate sized VTs. Further, it raises the possibility that energy failure may be a major contributor to eventual CPAP failure in these infants. That is, the energy requirement to maintain spontaneous breathing on CPAP eventually becomes too great.

In preterm infants high chest wall compliance combined with a low lung compliance increases the risk of inward chest wall distortion.[37] Low lung compliance and CPAP failure is contributed to by numerous factors such as lung structural immaturity, absence of surfactant and low lung gas volume due to partially liquid-filled lungs.[38–40] The contribution of lung tissue mechanics in CPAP failure is inconclusive; studies have found that infants who received antenatal steroids were less likely to fail CPAP [8, 11] whereas others report no significant relationship between antenatal steroids and CPAP failure [1, 9, 10]. In our study there was no significant difference in corticosteroid administration between CPAP-fail and CPAP-success infants. Stable micro bubble tests or lamella body count, which indirectly assesses the function of pulmonary surfactant, have shown to be a potential predictor of moderate to severe RDS.(12,13) Considering that 70% of newborns that fail CPAP have RDS and 53% demonstrate a severe form on chest X-rays [1], surfactant deficiency may play a role in CPAP failure by promoting low lung compliance. Alternatively, inadequate lung liquid clearance at birth decreases lung compliance. As lung liquid clearance is promoted by increased transpulmonary hydrostatic pressures gradients [14,16], successful lung recruitment at birth, possibly with the use of sustained inflations, could reduce the risk of CPAP-failure [9] by increasing lung compliance and achieving a lower chest wall to lung compliance ratio.

Previous research suggested that EHs are related to lung gas volume; newborn rabbits commonly utilised EHs after >50% of FRC had been accumulated [14], 1–2 week old lambs increased vagal afferent feedback and inhibited EHs when FRC was increased [23] and increasing CPAP in 1–21 day old infants increased FRC and reduced the frequency of EHs [22]. In our study the use of EH increased after birth, similar to that observed in rabbit pups [14], however it did not differ between CPAP-fail and CPAP-success infants. EHs likely do not reflect absolute lung gas volumes but rather relative changes in end-expiratory lung gas volumes. Although lung gas volumes were not measured in our study, CPAP-fail infants were likely to have had lower lung gas volumes than CPAP-success infants and indeed CPAP-fail infants required greater respiratory support. Increases and decreases in lung gas volume are not uncommon immediately after birth.[14,41] Therefore, each time end-expiratory lung gas volumes decrease, irrespective of original or subsequent lung gas volumes, vagal afferent feedback triggers the use of EHs to restore FRC.[42] Therefore, it would be difficult to differentiate between CPAP-success and CPAP-fail infants based on EH usage alone.

One of the major limitations to this study is the short duration of the respiratory recordings analysed. This was limited to the time that the infant is on the resuscitation table; in our study this ranged between 5 to 12 minutes. Within this short recording period, infant’s initiated spontaneous breathing and received IPPV at different times. The quality of the respiratory traces were affected by face mask leak and physical movement from the infant caused by positioning the infant into polyethylene wrap or placing the pulse oximeter probe on the infant’s wrist. These events further reduced the amount of analysable recording and may have prevented statistical differences from being detected between groups. If recordings were extended or performed later when the infant was quietly breathing and not interfered with by caregivers, clearer differences may have been detected. Indeed, in our study, infants were intubated at an average of 6 hours after birth. Infants intubated in the delivery room (~10%) were excluded, which would have removed the sickest infants from our analysis. However, we believed that this exclusion was necessary because our aim was to determine which infants could be stabilised with CPAP in the delivery room but require more intensive respiratory support later on.

## Conclusion

The ability to predict infants who will fail CPAP soon after birth is a valuable tool that would help caregivers to initiate treatments and preventative strategies early. This would prevent the infant from being intubated and mechanical ventilated throughout their recovery. Our study suggests that infants most likely to later fail CPAP are those who have weaker respiratory efforts at 9–12 minutes after birth. These infants more infrequently utilised high peak inspiratory flows, required increased peak inspiratory flows to generate moderately sized VTs and were unable to generate rapid peak inspiratory flows to perform recruitment manoeuvres (VTs >9 ml/kg). The difficulty the CPAP-fail infants face to maintain lung aeration immediately after birth is reflected in their greater need for respiratory support at birth before being transferred to the NICU. Further research is required to determine if it is feasible to determine the relationship between VT and inspiratory flow soon after birth or if determining the threshold of different forms of respiratory support will more easily identify infants most likely to fail CPAP.

## References

[1] Ammari A, Suri M, Milisavljevic V, Sahni R, Bateman D, Sanocka U et al Variables associated with the early failure of nasal CPAP in very low birth weight infants. J Pediatr 2005; 147: 341–7. 16182673 10.1016/j.jpeds.2005.04.062

[2] Morley CJ, Davis PG, Doyle LW, Brion LP, Hascoet JM, Carlin JB et al Nasal CPAP or intubation at birth for very preterm infants. N Engl J Med 2008; 358: 700–8. 10.1056/NEJMoa072788 18272893

[3] Finer NN, Carlo WA, Walsh MC, Rich W, Gantz MG, Laptook AR et al Early CPAP versus surfactant in extremely preterm infants. N Engl J Med 2010; 362: 1970–9. 10.1056/NEJMoa0911783 20472939 PMC3071534

[4] Sandri F, Plavka R, Ancora G, Simeoni U, Stranak Z, Martinelli S, et al Prophylactic or early selective surfactant combined with nCPAP in very preterm infants. Pediatrics 2010; 125: e1402–9. 10.1542/peds.2009-2131 20439601

[5] Dunn MS, Kaempf J, de Klerk A, de Klerk R, Reilly M, Howard D, et al Randomized trial comparing 3 approaches to the initial respiratory management of preterm neonates. Pediatrics 2011; 128: e1069–76. 10.1542/peds.2010-3848 22025591

[6] Lopez ES, Rodriguez EM, Navarro CR, Sanchez-Luna M. Initial respiratory management in preterm infants and bronchopulmonary dysplasia. Clinics (Sao Paulo) 2011; 66: 823–7. 21789387 10.1590/S1807-59322011000500019 PMC3109382

[7] Yost CC, Soll RF. Early versus delayed selective surfactant treatment for neonatal respiratory distress syndrome. Cochrane Database Syst Rev 2000: CD001456. 10.1002/14651858.CD001456 10796266

[8] Dimitriou G, Fouzas S, Giannakopoulos I, Papadopoulos VG, Decavalas G, Mantagos S. Prediction of respiratory failure in late-preterm infants with respiratory distress at birth. Eur J Pediatr 2011; 170: 45–50. 10.1007/s00431-010-1264-x 20669031

[9] Fuchs H, Lindner W, Leiprecht A, Mendler MR, Hummler HD. Predictors of early nasal CPAP failure and effects of various intubation criteria on the rate of mechanical ventilation in preterm infants of <29 weeks gestational age. Arch Dis Child Fetal Neonatal Ed 2011; 96: F343–7. 10.1136/adc.2010.205898 21278432

[10] De Jaegere AP, van der Lee JH, Cante C, van Kaam AH. Early prediction of nasal continuous positive airway pressure failure in preterm infants less than 30 weeks gestation. Acta Paediatr 2012; 101: 374–9. 10.1111/j.1651-2227.2011.02558.x 22150698

[11] Pillai MS, Sankar MJ, Mani K, Agarwal R, Paul VK, Deorari AK et al Clinical prediction score for nasal CPAP failure in pre-term VLBW neonates with early onset respiratory distress. J Trop Pediatr 2011; 57: 274–9. 10.1093/tropej/fmq047 20558382

[12] Daniel IWBD, Fiori HH, Piva JP, Munhoz TP, Nectoux AV, Fiori RM. Lamellar Body Count and Stable Microbubble Test on Gastric Aspirates from Preterm Infants for the Diagnosis of Respiratory Distress Syndrome. Neonatology 2010; 98: 150–5. 10.1159/000279887 20234139

[13] Fiori HH, Fritscher CC, Fiori RM. Selective surfactant prophylaxis in preterm infants born at < or = 31 weeks' gestation using the stable microbubble test in gastric aspirates. J Perinat Med 2006; 34: 66–70. 16489887 10.1515/JPM.2006.008

[14] Siew ML, Wallace MJ, Kitchen MJ, Lewis RA, Fouras A, te Pas AB, et al Inspiration regulates the rate and temporal pattern of lung liquid clearance and lung aeration at birth. J Appl Physiol 2009; 106: 1888–95. 10.1152/japplphysiol.91526.2008 19342434

[15] Hutchison AA, Wozniak JA. Endotracheal measurement of thyroarytenoid activity in newborn lambs. Biol Neonat 2000; 78: 139–44. 10971007 10.1159/000014262

[16] Hooper SB, Kitchen MJ, Wallace MJ, Yagi N, Uesugi K, Morgan MJ et al Imaging lung aeration and lung liquid clearance at birth. FASEB J 2007; 21: 3329–37. 17536040 10.1096/fj.07-8208com

[17] Gerhardt T, Bancalari E. Chestwall compliance in full-term and premature infants. Acta Paediatr 1980; 69: 359–64. 7376862 10.1111/j.1651-2227.1980.tb07093.x

[18] Fisher JT, Mortola JP, Smith JB, Fox GS, Weeks S. Respiration in Newborns—Development of the Control of Breathing. American Review of Respiratory Disease 1982; 125: 650–7. 7091870 10.1164/arrd.1982.125.6.650

[19] te Pas AB, Wong C, Kamlin COF, Dawson JA, Morley CJ, Davis PG. Breathing Patterns in Preterm and Term Infants Immediately After Birth. Pediatr Res 2009; 65: 352–6. 10.1203/PDR.0b013e318193f117 19391251

[20] te Pas AB, Davis PG, Kamlin CO, Dawson J, O'Donnell CP, Morley CJ. Spontaneous breathing patterns of very preterm infants treated with continuous positive airway pressure at birth. Pediatr Res 2008; 64: 281–5. 10.1203/PDR.0b013e31817d9c35 18458652

[21] Kosch PC, Stark AR. Dynamic Maintenance of End-Expiratory Lung-Volume in Full-Term Infants. J Appl Physiol 1984; 57: 1126–33. 6501029 10.1152/jappl.1984.57.4.1126

[22] Elgellab A, Riou Y, Abbazine A, Truffert P, Matran R, Lequien P et al Effects of nasal continuous positive airway pressure (NCPAP) on breathing pattern in spontaneously breathing premature newborn infants. Intensive Care Med 2001; 27: 1782–7. 11810123 10.1007/s00134-001-1117-1

[23] Harding R. State-related and developmental changes in laryngeal function. Sleep 1980; 3: 307–22. 7221340 10.1093/sleep/3.3-4.307

[24] Vyas H, Field D, Milner AD, Hopkin IE. Determinants of the first Inspiratory Volume and Functional Residual Capacity at Birth. Pediatr Pulmonol 1986; 2: 189–93. 3763256 10.1002/ppul.1950020403

[25] Mortola JP, Milicemili J, Noworaj A, Smith B, Fox G, Weeks S. Muscle Pressure and Flow During Expiration in Infants. American Review of Respiratory Disease 1984;129:49–53. 6703485 10.1164/arrd.1984.129.1.49

[26] Frappell PB, MacFarlane PM. Development of mechanics and pulmonary reflexes. Respiratory Physiology & Neurobiology 2005; 149: 143–54. 16046198 10.1016/j.resp.2005.05.028

[27] Dargaville PA, Aiyappan A, De Paoli AG, Dalton RG, Kuschel CA, Kamlin CO et al Continuous Positive Airway Pressure Failure in Preterm Infants: Incidence, Predictors and Consequences. Neonatology 2013;104:8–14. 10.1159/000346460 23595061

[28] Schilleman K, Siew ML, Lopriore E, Morley CJ, Walther FJ, te Pas AB. Auditing resuscitation of preterm infants at birth by recording video and physiological parameters. Resuscitation 2012;83:1135–9. 10.1016/j.resuscitation.2012.01.036 22322286

[29] Richtlijn Reanimatie van pasgeborene, NVK. http://www.nvk.nl/tabid/1558/articleType/ArticleView/articleId/765/default.aspx

[30] Schmolzer GM, Kamlin OCOF, Dawson JA, Te Pas AB, Morley CJ, Davis PG. Respiratory monitoring of neonatal resuscitation. Arch Dis Child Fetal Neonatal Ed 2010; 95: F295–F303. 10.1136/adc.2009.165878 19776023

[31] Khan N, Brown A, Venkataraman ST. Predictors of extubation success and failure in mechanically ventilated infants and children. Crit Care Med 1996; 24: 1568–79. 8797633 10.1097/00003246-199609000-00023

[32] Kaczmarek J, Kamlin COF, Morley CJ, Davis PG, Sant'Anna GM. Variability of respiratory parameters and extubation readiness in ventilated neonates. Arch Dis Child Fetal Neonatal Ed 2013; 98: F70–3. 10.1136/fetalneonatal-2011-301340 22556206

[33] Bien MY, Lin YS, Shih CH, Yang YL, Lin HW, Bai KJ et al Comparisons of predictive performance of breathing pattern variability measured during T-piece, automatic tube compensation, and pressure support ventilation for weaning intensive care unit patients from mechanical ventilation. Crit Care Med 2011; 39: 2253–62. 10.1097/CCM.0b013e31822279ed 21666447

[34] Bushell T, McHugh C, Meyer MP. A comparison of two nasal continuous positive airway pressure interfaces—a randomized crossover study. J Neonatal Perinatal Med. 2013; 6: 53–9. 10.3233/NPM-1363612 24246459

[35] Gaultier C. Respiratory muscle function in infants. Eur Respir J 1995; 8: 150–3. 7744181 10.1183/09031936.95.08010150

[36] Warren RH, Horan SM, Robertson PK. Chest wall motion in preterm infants using respiratory inductive plethysmography. Eur Resp J 1997; 10: 2295–2300. 9387956 10.1183/09031936.97.10102295

[37] Heldt GP, McIlroy MB. Distortion of chest wall and work of diaphragm in preterm infants. J Appl Physiol 1987; 62: 164–9. 3558176 10.1152/jappl.1987.62.1.164

[38] Siew ML, te Pas AB, Wallace MJ, Kitchen MJ, Islam MS, Lewis RA et al Surfactant increases the uniformity of lung aeration at birth in ventilated preterm rabbits. Pediatr Res 2011;70:50–55. 10.1038/pr.2011.275 21451432

[39] Kelly E, Bryan H, Possmayer F, Frndova H, Bryan C. Compliance of the respiratory system in newborn infants pre- and postsurfactant replacement therapy. Pediatr Pulmonol 1993; 15: 225–30. 8469575 10.1002/ppul.1950150408

[40] Nilsson R, Grossmann G, Robertson B. Artificial ventilation of premature newborn rabbits: effects of positive end-expiratory pressure on lung mechanics and lung morphology. Acta Paediatr 1980; 69: 597–602. 7015781 10.1111/j.1651-2227.1980.tb07328.x

[41] Mortola JP, Gisher JT, Smith JB, Fox GS, Weeks S, Willis D. Onset of respiration in infants delivered by cesarean section. J Appl Physiol 1982; 52: 716–24. 7068487 10.1152/jappl.1982.52.3.716

[42] Wong KA, Bano A, Rigaux A, Wang B, Bharadwaj B, Schürch S et al Pulmonary vagal innervation is required to establish adequate alveolar ventilation in the newborn lamb. J Appl Physiol 1998; 85: 849–59. 9729557 10.1152/jappl.1998.85.3.849

